# Effect of 5-Minute Movies Shown via a Mobile Phone App on Risk Factors and Mortality After Stroke in a Low- to Middle-Income Country: Randomized Controlled Trial for the Stroke Caregiver Dyad Education Intervention (Movies4Stroke)

**DOI:** 10.2196/12113

**Published:** 2020-01-28

**Authors:** Ayeesha Kamal, Adeel Khoja, Bushra Usmani, Shahvaiz Magsi, Aresha Malani, Zahra Peera, Saadia Sattar, Masood Ahmed Akram, Sumaira Shahnawaz, Maryam Zulfiqar, Abdul Muqeet, Fabiha Zaidi, Saleem Sayani, Azmina Artani, Iqbal Azam, Sarah Saleem

**Affiliations:** 1 Aga Khan University Stroke Services and Research Karachi Pakistan; 2 Aga Khan Development Network Digital Health Resource Center Karachi Pakistan; 3 Aga Khan University Community Health Sciences Karachi Pakistan

**Keywords:** stroke, mobile health, noncommunicable diseases, adherence

## Abstract

**Background:**

Pakistan is the sixth most populous nation in the world and has an estimated 4 million stroke survivors. Most survivors are taken care of by community-based caregivers, and there are no inpatient rehabilitation facilities.

**Objective:**

The objective of this study was to evaluate the effectiveness and safety of locally designed 5-min movies rolled out in order of relevance that are thematically delivered in a 3-month program to deliver poststroke education to stroke survivor and caregiver dyads returning to the community.

**Methods:**

This study was a randomized controlled, outcome assessor–blinded, parallel group, single-center superiority trial in which participants (stroke survivor-caregiver dyads) with first-ever stroke (both ischemic and hemorrhagic) incidence were randomized within 48 hours of their stroke into either the video-based education intervention group or the control group. The video-based education intervention group had health education delivered through short videos that were shown to the participants and their caregivers at the time of admission, before discharge, and the first and third months of follow-up after discharge. The control group had standardized care including predischarge education and counseling according to defined protocols. All participants enrolled in the video education intervention and control groups were followed for 12 months after discharge for outcome assessment in the outpatient stroke clinics. The primary outcome measures were the proportion of participants achieving control of blood pressure, blood sugar, and blood cholesterol in the video intervention versus the control group. Several predefined secondary outcomes were included in this study, of which we report the mortality and functional disability in this paper. Analysis was by performed using the intention-to-treat principle.

**Results:**

A total of 310 stroke survivors and their caregiver dyads (participant dyads) were recruited over a duration of 6 months. In total, 155 participant dyads were randomized into the intervention and control groups, each. The primary outcome of control of three major risk factors revealed that at 12 months, there was a greater percentage of participants with a systolic B*P*<125 mm Hg (18/54, 33% vs 11/52, 21%; *P*=.16), diastolic B*P*<85 mm Hg (44/54, 81% vs 37/52, 71%; *P*=.21), HbA_1c_ level<7% (36/55, 65% vs 30/40, 75%; *P*=.32), and low-density lipoprotein level<100 mg/dL (36/51, 70% vs 30/45, 67%; *P*=.68) in the intervention group than in the control group. The secondary outcome reported is the mortality among the stroke survivors because the number of stroke-related complications was higher in the control group than in the intervention group (13/155, 8.4% vs 2/155, 1.3%), and this difference was statistically significant (*P*<.001).

**Conclusions:**

The Movies4Stroke trial failed to achieve its primary specified outcome. However, secondary outcomes that directly related to survival skills of stroke survivors demonstrated the effectiveness of the video-based intervention on improving stroke-related mortality and survival without disability.

**Trial Registration:**

ClinicalTrials.gov NCT02202330; https://www.clinicaltrials.gov/ct2/show/NCT02202330

## Introduction

### Background

Stroke is the second leading cause of death globally and the principal cause of acquired disability in adults. About two-thirds of this burden is endured by the developing world [1].

Noncommunicable diseases (NCDs) are the biggest contributors to the rising incidence of stroke. Around 90.5% of the global stroke burden is attributable to modifiable risk factors, including 74.2% attributed to behavioral factors (smoking, poor diet, and low physical activity). In addition, hypertension, type 2 diabetes mellitus, and coronary artery disease are important modifiable risk factors for stroke [2]. Pakistan also has a disproportionate burden of stroke and NCD risk factors. At present, around 1 in 4 adult Pakistanis has hypertension or diabetes, heart disease, or a stroke equivalent, with most being unaware of their risks [3]. A local study investigated the prevalence of stroke in Pakistan among adult Pashtun population and reported a prevalence of 4.8%, which is equal to 4 million persons affected in a country with a population of 180 million [3].

Studies that describe the outcomes of stroke survivors in this setting report that at a median of 5.5 months after discharge, 12.3% of the patients had died, mostly from recurrent vascular events or stroke complications. Poor functional outcome, defined as Modified Rankin Scale (mRS) score>2, was seen in 51% of the study participants, and cognitive outcomes were poor in 42% of the survivors [4].

In a country of a population of 180 million, roughly only 23 centers exist to provide physical medicine or help with rehabilitation; most have not adopted a multidisciplinary approach toward patients, and none have inpatient services [5]. Currently, there are no organized home care survival programs involving primary caretakers for stroke survivors in Pakistan.

Despite these challenges, there is potential to leverage mobile technology to improve stroke outcomes. Pakistan has widespread mobile connectivity, with a cellular density of 77% [6]. These infrastructure enablers create distinct opportunities for mobile health (mHealth). Our rationale was to leverage information technology (IT)–based mHealth to provide a solution and knowledge and direct skills to the survivor and caregiver where the provision of chronic care is rudimentary.

### Objective

In this study, we aimed to evaluate the effectiveness and safety of locally designed 5-min movies rolled out in order of relevance that are thematically delivered in a 3-month program to deliver poststroke education to stroke survivor and caregiver dyads returning to the community. We hypothesized that the absence of trained personnel in the health community could be mitigated by actually providing high-quality repetitive training using audio visual aids that served as a checklist for competency and survival skills to the stroke survivor and caregiver dyad [7,8].

## Methods

### Study Design

A randomized controlled, outcome assessor–blinded, parallel group, single-center superiority trial was conducted to assess the efficacy of mobile phone video–based IT intervention for controlling 3 major risk factors–blood pressure [BP], blood glucose, and cholesterol—among adult stroke survivors. Important secondary outcomes included postdischarge mortality attributable to stroke and measures of functional disability. Our detailed protocol has been published separately [9].

### Study Site

This trial was conducted in the Stroke unit, Neurology Ward, Aga Khan University Hospital (AKUH), Karachi, Pakistan. AKUH is an internationally recognized tertiary care institution, certified by Joint Commission International Accreditation, and caters to the needs of a large multiethnic urban population of 18 million people. Stroke care follows international protocols, with defined order sets and standardized pathways.

### Participants

The sample population comprised adults (aged>18 years) admitted to AKUH with first-ever acute stroke and having a designated caregiver, meeting the eligibility criteria, and giving informed consent.

The criteria used while recruiting participants and caregivers at the initial phase of the selection process are listed below.

### Eligibility Criteria

The eligibility criteria have been presented in Textboxes 1 and 2.

**Inclusion criteria.**

- Adult men and women aged ≥18 years of age
- Residents of Karachi and planning to live in Karachi till the follow-up period
- Able to understand Urdu (language of the videos) and the national language
- Admitted with first-ever stroke (ischemic or hemorrhagic)
- Modified Rankin Scale score≤4 (mild to moderate disability)
- Having at least one vascular risk factor that requires medical intervention
- Consenting to participate in the study and for follow-up visits, both stroke survivor and caregiver
- Have a designated caregiver at home who is responsible for appointments, follow-up, and overall care and are mobile phone literate, for example, wife, daughters, daughter-in-law, and husband
- Stroke was medically stable, and participant was likely to return to the community after the in-hospital stay (thus actively treated strokes such as decompressive surgeries, carotid endarterectomy, in-hospital sepsis, and ventilator complications that essentially preclude return to the community settings were not offered in this chronic care support study).

**Exclusion criteria.**

- Serious aphasia, visual hemineglect, short-term memory loss in the stroke survivor precluding understanding, visualization, or retention of the video material.
- Serious aphasia, visual hemineglect, short-term memory loss, dementia in the caregiver precluding understanding, visualization, or retention of the video material.
- Iatrogenic stroke, that is, stroke due to nonatherosclerotic vascular disease and rare causes, for example, carotid dissections, gunshot to neck, and coronary artery bypass surgery
- Stroke survivor/caregiver dyad continued poststroke care in a nursing-assisted, professional, or hospital setting and does not return to the community after discharge
- Serious concurrent medical illnesses such as cancer, renal failure, acute liver disease in past 6 months (that precludes use of statins), and chronic liver disease, which that exclude the use of stroke preventive medications or require nonstandardized therapy
- Any use of off-label, nonguideline medications, because of the stroke survivors’ unique comorbidities, that interferes with medication compliance to antihypertensive, statins, antiplatelet, and antidiabetic agents

### Randomization Process

Stroke survivors and their caregivers (dyad) were assigned to either the intervention or the usual care (control) groups in a parallel manner in a ratio of 1:1. Block randomization technique with a fixed block size of 10 was used. A computer-generated randomization list was used to randomize participants into the intervention or control group. The randomization center was performed in a secure computer in the clinical trials unit (CTU), and the randomization list was generated by CTU staff not involved in recruitment, outcome ascertainment, or any aspect of the study.

### Allocation Concealment

The randomization list was centralized and thus not predictable. No one from the research team had any access to randomization list, randomization envelopes, and block size or code. Envelopes were sealed and opaque, and it was impossible to view the sequence even if held against bright sunlight. Randomization list and opaque envelopes containing the randomization sequence were always kept inside the premises of CTU under lock and key.

### Identification and Enrollment of the Study Participants

Informed consent was obtained from eligible participants who volunteered to be a part of this study after they were provided a thorough explanation regarding the nature of the study and the scheduled follow-up visits. A detailed face-to-face interview of stroke survivors and their caregivers was conducted to gather data on sociodemographic and medical history. A baseline clinical and functional assessment was performed, after which they were randomly assigned into the intervention or control group by a trained research officer who was not blinded to the assignment of the intervention. Details regarding the proper functioning of mobile app and its installation were taught to the participants in the intervention group. A memory card containing Movies4Stroke app was installed in the participant’s Android phone along with the delivery of the first set of 5-min videos. Videos were shown at the time of enrollment into the study, at discharge, and at the first and third month after discharge from the hospital. The app was designed to provide access to the videos in a scheduled manner. To maintain contact and follow-up, a Stroke Helpline number was provided to participants in both the groups. The helpline number was active 24/7, and the person operating at the helpline number was trained to receive calls with the most frequently anticipated questions and to answer them accordingly. The operator had access to a stroke specialist at all times for support. If the participant was allocated to the usual care group, he/she was informed about the details regarding discharge, follow-up appointments at the clinic, and access to free lab vouchers at the 6th and 12th months.

### Sampling Technique

A purposive sample was selected from adult stroke survivors admitted into the Stroke unit, Neurology Ward, AKUH, Karachi, after the assessment of the eligibility criteria and obtaining informed consent.

### Technical Part of the Intervention

The Movies4Stroke app was developed by biomedical and software engineers of Aga Khan Development Network Electronic Health (eHealth) Resource Center in collaboration with stroke specialists, rehabilitation and swallowing experts, and epidemiologists. The intervention was first pilot tested on study team members’ Android cell phones. Any discrepancies and bugs were removed from the app. The intervention was then launched on tablets, specially purchased for showing movies to the stroke survivors and their caregivers in clinical ward settings. Memory chips were also purchased, so that the Movies4Stroke app could be transferred into the participants’ cell phones.

Participants in the intervention group were sent weekly messages twice as a reminder to watch the movies at home. These messages were sent through a Web-based, programmed, open-access software entitled *Frontline* by a trained IT professional.

### Intervention Group

**Figure 1:**
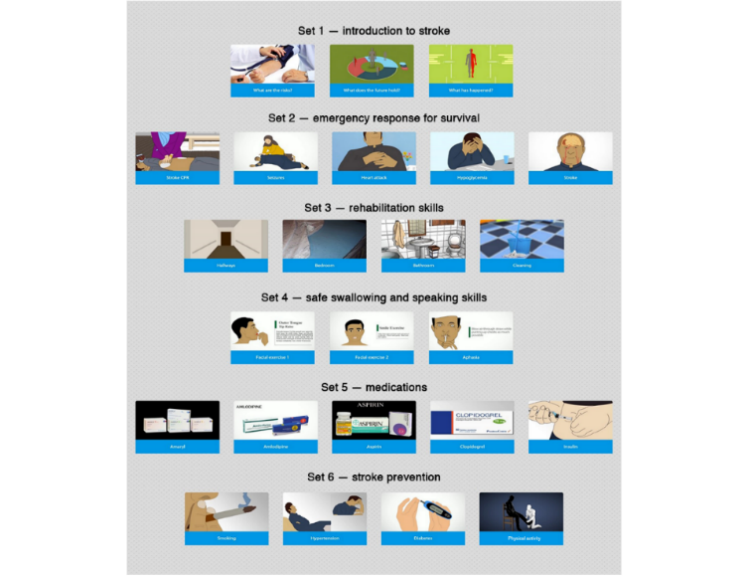
Movies4Stroke collage. CPR: cardiopulmonary resuscitation.

In addition to the usual care, the intervention group at the time of admission received the introductory teaching session with installation of the app and the first set of 5-min videos on various stroke-related topics as described below. In the first session, different skills such as swallowing exercises, different rehabilitation exercises, and nasogastric tube feeding were taught to the caregivers. The second session was delivered at the time of discharge, which included videos on emergency preparedness, such as cardiopulmonary resuscitation, seizures, heart attack, and hypoglycemia, while simultaneously discussing and answering any queries the participants had after watching each set of videos. The third session was delivered at the first month of follow-up after discharge and included videos on frequently used medications by stroke survivors, such as anticoagulants, antihypertensive, and lipid-lowering drugs. The fourth session was delivered at the third-month of follow-up after discharge and included videos on secondary stroke prevention (recurrent attack)—exercise, physical activity, recognition of depression, diet modification, and accurate measurement of BP and blood sugar levels (Figure 1).

### Control Group

Participants in the control group received the standard of care that is provided to stroke patients at AKUH. Stroke survivors were given instructions before discharge regarding diet, the need for rehabilitation, possible complications, and medication use; information booklets were also handed out. A multidisciplinary team comprising a neurophysician, stroke nurse, dietitian, and physiotherapist imparted the information. Verbal instructions were given to stroke survivors and their caregivers. On the day of discharge, or 24 hours before discharge, a discharge coordinator provided the researchers details about the skills learned and ensured that the medical, social, and rehabilitation requirements were in place before going out of the hospital. All the study participants were provided follow-up appointments at the clinic. A detailed written discharge summary was handed over to the caregiver, detailing all aspects of care, including follow-up visit, medications, lab investigations, and serious alerts. The control group compliance to this standard of care was ensured, as all staff follow the abovementioned discharge protocol and document education. There is designated staff education and a safe discharge coordinator dedicated for all admissions at this center. The center at which this study was performed is an internationally accredited center, and performance and documentation of these quality of care standards are a part of the standard of care protocol. This standard of care was followed for all participants in the control group because of these regulations that are in place to maintain accreditation and auditable quality of care. The control group did not receive the additional visual teaching of the video-based intervention. This standard of care was followed for all participants including those who received the video-based intervention.

### Compliance During the Administration of the Intervention

A study officer who was not blinded to the intervention group took several measures to ensure compliance of the participants to the videos as mentioned in the protocol [9]. There were trained research officers to ensure compliance of the intervention group participants at each video delivery; moreover, constant SMS reminders were sent to the study participants in the intervention group (as a measure of reinforcement) to watch these thematic movies in a relaxed home environment, and they were also reminded about their scheduled follow-up visits.

### Follow-Up

Follow-up visit for each stroke survivor-caregiver dyad was organized at 1, 3, 6, 9, and 12 months after discharge in the neurology clinic for outcome ascertainment. Stroke survivors and their caregivers were given a handout with instructions and basic information about their subsequent follow-up visit. Caregivers were also explained verbally about the importance of their follow-up visit. They were asked to contact the study team through the Stroke Helpline for any queries regarding their health.

**Figure 2:**
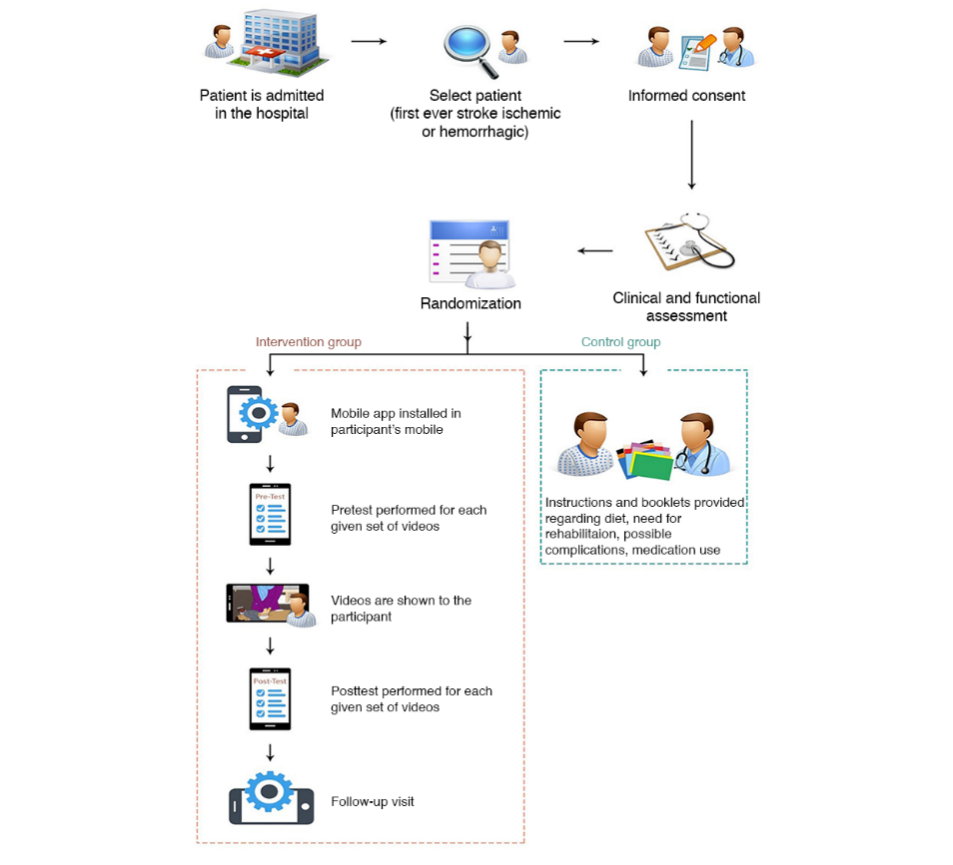
Study flow (enrollment to follow-up).

The follow-up rates were maximized by sending SMS reminders to all the study participants about their respective follow-up visits at least a day before their scheduled follow-up visit through our Stroke Helpline number and by also allowing an approximate 14-day grace period to the stroke survivor and caregiver who were unable to report as per their scheduled follow-up visit. Those participants who did not appear for their scheduled follow-up visit were contacted through phone or approached through indirect means, such as contacting them when they came to the AKUH for any other clinic or physiotherapy visit or lab investigations. Details of stroke survivors’ visit to the AKUH, other than the neurology clinic, was obtained through telecommunication with the caregiver or tracking the stroke survivor through the synchronized electronic medical record system of the AKUH (Figure 2).

### Participants’ Timeline

After recruiting stroke survivors along with their primary caregivers from the Stroke unit at the AKUH, the participant dyads were not expected to come in for any additional visits for the study purpose other than the scheduled 5 follow-up visits at the stroke clinic. Our study started enrolling participants from January 19, 2015, and the last participant was recruited on May 15, 2015. The last follow-up was completed on June 29, 2016.

### Trial Outcomes

The primary outcome measure reported was as follows: control of 3 major risk factors—BP, blood sugar, and lipids—measured via standardized methods in the central laboratory was ascertained at baseline and 6 and 12 months after discharge. All the 3 risk factors were assessed, as the proportion of participants achieving BP control (<125/85 mm Hg), blood sugar (glycosylated hemoglobin A_1c_ or HbA_1c_<7%), and blood cholesterol (low-density lipoprotein [LDL] level<100 mg/dL).

Of the secondary outcomes, two are discussed in this paper with respect to Movies4Stroke trial protocol; others will be discussed in the subsequent paper.

### Stroke-Related Mortality Among Stroke Survivors Was Ascertained at 12 Months Post Discharge

Information on mortality among stroke survivors after discharge was ascertained through a precoded and validated verbal autopsy scale. In addition, we correlated all mortality with hospital records. Mortality was further categorized as per the analysis criteria into 3 categories: stroke-related mortality, mortality (because of nonstroke complications), and out-of-hospital mortality.

#### Included

Mortality after discharge from the AKUH because of stroke-related complications was assessed by a review of medical records, interviews from primary caregivers of the patients, and the verbal autopsy standard procedure.

#### Censored

Mortality after discharge from the hospital because of competing risk, that is, cause of death not related to stroke because of, for example, head trauma, gun shot, and cancer, was assessed.

Patients who were alive after 1 year of follow-up after discharge (censored because of lack of outcome of interest or statistical considerations) were assessed.

#### Excluded

In-hospital mortality before patients were discharged from the hospital was a result of complications arising from an index stroke occurring before discharge into the community in a stable state, for example, iatrogenic complications, sepsis, and progression of index stroke during admission.

Moreover, the categorization of all these deaths was further validated by our team of experts including stroke specialist, epidemiologist, statisticians, and research supervisor.

### Stroke Disability and Neurological Deficits Among Stroke Survivors Was Ascertained at Baseline and 6 and 12 Months After Discharge

A total of 3 different neurological functional assessment tools were used in this clinical trial, as each of them captures/measures a different parameter with respect to the functional status of stroke survivors after acute stroke:

- mRS tool is widely used by neuro physicians globally to assess functional disability after acute stroke [10-12].
- The National Institutes of Health Stroke Scale (NIHSS) tool is widely used by neurophysicians globally to objectively quantify the impairment caused by stroke [12,13].
- The Barthel Index (BI) tool is used globally by health care providers to assess the level of dependency among stroke survivors after an acute episode of stroke [12,14].

The abovementioned tools were used to assess the functional status of stroke survivors at baseline and 6- and 12-month follow-ups.

### Ethics and Human Subject Protection

Written informed consent, in both English and Urdu, was obtained from all the study participants at the time of recruitment. The confidentiality and privacy of the participants was maintained by deidentification of the subject information. Only research staff was authorized entry into the hospital system on the computers that were used for data storage. All source documents were maintained in locked files in locked room. Fingerprint encryption was added to all sensitive data, for example, mobile numbers, app logs, and error logs. The Ethical Review Committee (ERC) of Aga Khan University, Karachi (ERC number 2890-Med-ERC-14), approved the study.

## Results

### Overall Trial Flow

**Figure 3:**
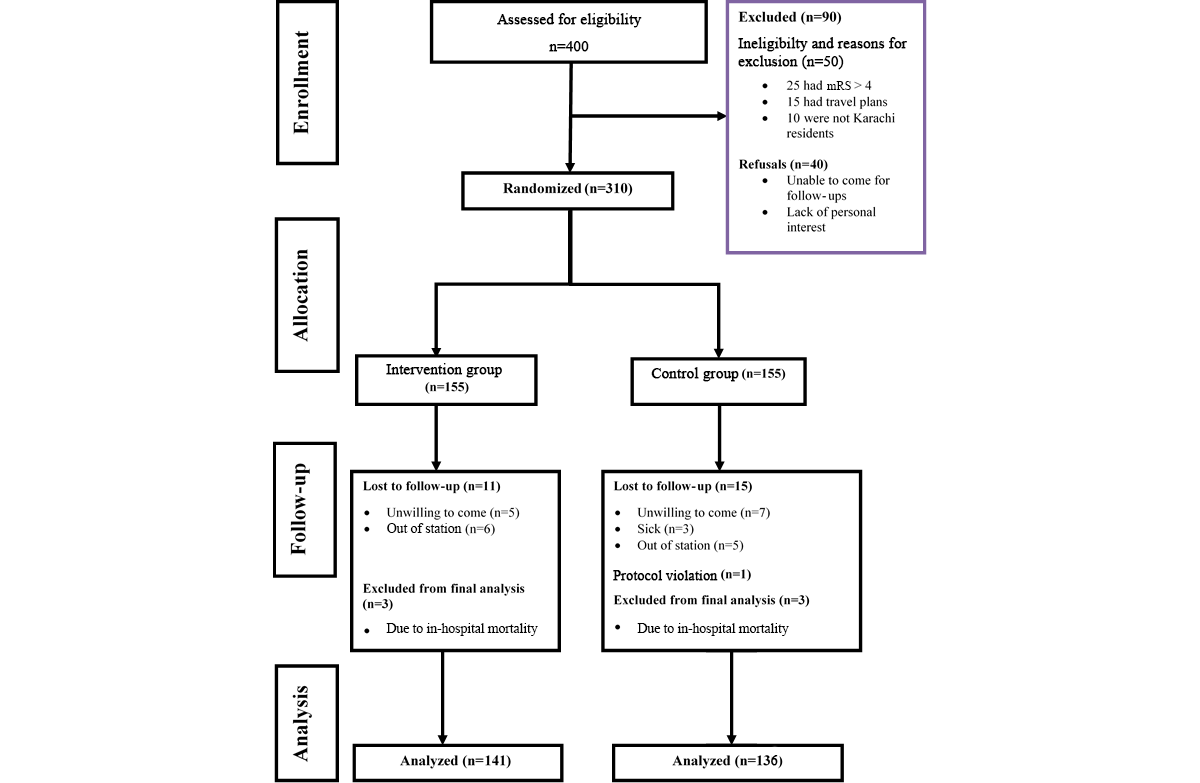
Trial flow diagram. mRS: Modified Rankin Scale.

A total of 310 stroke survivors and their caregiver dyads, ie, 620 individuals (participant dyads), were recruited over a duration of 6 months. As this clinical trial had a fixed block design, 155 participant dyads were randomized in each of the intervention and control group (310 in each group). We screened 400 participant dyads to assess eligibility; of these, 50 were not eligible, and 40 participant dyads refused to participate in the study (30% were excluded; Figure 3). The reasons for exclusions were mRS>4 (n=25), travel plans (n=15), and non-Karachi residents (n=10). The reasons for refusal were mainly the lack of ability to return for follow-up and personal interest of the stroke survivor or caregiver to participate in the study. We were able to complete information on 141 participant dyads in the intervention and 137 in the control group at the end of 1-year postdischarge follow-up. From the intervention group, 11 participant dyads were lost to follow-up at 1 year postdischarge and 3 stroke survivors died because of in-hospital mortality (before being discharged) resulting from inpatient complications from the index stroke, as compared with 15 participant dyads who were lost to follow-up and 3 who died because of in-hospital mortality in the control group. There was one protocol violation in the control group that was excluded from the final analysis.

### Baseline Characteristics of the Study Participants

Mean age of stroke survivors in the intervention group was 60.6 (SD 12.0) years, whereas it was 59.7 (SD 14.3) years in the control group. The caregivers were relatively younger, with the mean age of 38.7 (SD 11.7) years in the intervention group and 39.8 (SD 14) years in the control group. In our trial, most of the stroke survivors were males (109/155, 70.3%, in the intervention group vs 100/155, 65.0%, in the control group). More than two-thirds of the stroke survivors in our study had more than 5 years of education (114/155, 73.5%, in the intervention group vs 107/155, 69.0%, in the control group), with 40 of 155 (25.8%) patients being employed in the intervention group and 39 of 155 (25.1%) in the control group. More than four-fifths of our study participants were married (134/155, 86.5%) in the intervention group as compared to the control group (124/155, 80.0%), and more than half of the stroke survivors were living in a joint family system (93/155, 60.0%, in the intervention group vs 107/155, 69.0%, in the control group). Median (interquartile range) number of household members living with stroke survivors was 6 (range: 5-8) in the intervention group as compared with 7 (range: 5-10) in the control group. Median household monthly income of the stroke survivors was Rs 50,000 (US $416) in both the groups. Approximately, four-fifths of our study participants were Android mobile phone users in the intervention group (120/155, 77.4%) as compared with around two-third in the control group (99/155, 63.8%). Most variables were uniformly distributed between the two groups and not statistically significant at baseline (refer to Table 1).

**Table 1:** Baseline characteristics of study participants according to their group allocation (310 participant dyads)

Baseline characteristics		Intervention group (N=155)	Control group (N=155)	*P* value
Age (years) of the patient, mean (SD)		60.6 (12.0)	59.7 (14.3)	.17
**Patient’s gender, n (%)**				.35
	Male	109 (70.0)	100 (65.0)	
	Female	46 (30.0)	55 (35.0)	
Age (years) of the caregiver, mean (SD)		38.7 (11.7)	39.8 (14.0)	
**Source of the patient, n (%)**				.26
	Direct	90 (58.0)	81 (52.2)	
	Referral	65 (42.0)	74 (47.8)	
**Type of operating system, n (%)**				.01
	None	14 (9.0)	30 (19.3)	
	Android	127 (82.0)	104 (67.1)	
	Windows	6 (3.9)	13 (8.4)	
	IOS	8 (5.1)	8 (5.2)	
**Anyone at home with Android, n (%)**				<.001
	Yes	120 (77.4)	99 (63.8)	
	No	35 (22.6)	56 (36.2)	
**Desktop PC at home, n (%)**				.02
	Yes	143 (92.3)	130 (83.8)	
	No	12 (7.7)	25 (16.2)	
**Patient education, n (%)**				.22
	Illiterate	17 (11.0)	31 (20.0)	
	Primary education	24 (15.5)	17 (11.0)	
	Secondary education	36 (23.2)	34 (22.0)	
	Higher secondary education	21 (13.5)	18 (11.6)	
	Above intermediate	57 (36.8)	55 (35.4)	
**Patient marital status, n (%)**				.14
	Single	3 (1.9)	9 (5.8)	
	Married	134 (86.5)	124 (80.0)	
	Widowed	18 (11.6)	22 (14.2)	
**Patient family status, n (%)**				.12
	Joint family	93 (60.0)	107 (69.0)	
	Nuclear family	62 (40.0)	48 (31.0)	
Monthly family income (Pakistani rupee), median (IQR)		50,000 (30,000-100,000)	50,000 (30,000-70,000)	
**Patient employment status, n (%)**				.45
	Employed	40 (25.8)	39 (25.1)	
	Unemployed	5 (3.3)	10 (6.5)	
	Retired	27 (17.4)	27 (17.4)	
	Housewife	43 (27.7)	50 (32.2)	
	Others	40 (25.8)	29 (18.7)	
**Family land ownership (acre), n (%)**				.92
	None	47 (30.3)	47 (30.3)	
	<1	92 (59.4)	89 (57.4)	
	Between 1 and 10	10 (6.4)	13 (8.4)	
	>10	6 (3.9)	6 (3.9)	
Total household members, median (IQR)		6 (5-8)	7 (5-10)	.52
Length of hospital stay, median (IQR)		4 (3-4)	3 (3-5)	.71
**Tissue plasminogen activator, n (%)**				.98
	Yes	7 (4.5)	7 (4.5)	
	No	148 (95.5)	148 (95.5)	

### Primary Outcome

#### Systolic Blood Pressure

At baseline, there was a uniform distribution of study participants with the two categories of systolic BP (<125 mm Hg and >125 mm Hg) in the intervention group and the control group (49/155, 50.0%, vs 48/155, 50.0%). At the 6-month visit, the distribution of study participants with a systolic BP of <125 mm Hg was similar in the intervention group and control group (36/109, 50.0% vs 36/99, 50.0%; risk ratio [RR] 0.91, 95% CI 0.62-1.32). At the final follow-up visit (at 12 months), there was a greater percentage of participants with a systolic BP of <125 mm Hg in the intervention group than in the control group (18/54, 62.0% vs 11/52, 38.0%; RR 1.58, 95% CI 0.83-2.98). However, none of these results were statistically significant (refer to Table 2).

**Table 2:** Systolic blood pressure.

Systolic blood pressure (mm Hg)		Intervention group, n (%)	Control group, n (%)	Risk ratio (95% CI)	*P* value (overall)
**Baseline results (N=310)^a^**					.96
	<125	49 (50.0)	48 (50.0)	1.00 (0.72-1.40)	
	>125	106 (50.0)	107 (50.0)	1.00 (0.72-1.40)	
**6-month results (N=208)^b^**					.61
	<125	36 (50.0)	36 (50.0)	0.91 (0.62-1.32)	
	>125	73 (54.0)	63 (46.0)	0.91 (0.62-1.32)	
**12-month results (N=106)^c^**					.16
	<125	18 (62.0)	11 (38.0)	1.58 (0.83-2.98)	
	>125	36 (47.0)	41 (53.0)	1.58 (0.83-2.98)	

^a^Baseline: intervention group (n=155) and control group (n=155).

^b^6-month results: intervention group (n=109) and control group (n=99).

^c^12-month results: intervention group (n=54) and control group (n=52).

#### Diastolic Blood Pressure

At baseline, there were slightly more participants with a diastolic BP of <85 mm Hg in the intervention group than in the control group (109/155, 51.0% vs 106/155, 49.0%). At 6 months, there was a greater percentage of stroke survivors with a diastolic BP of <85 mm Hg in the intervention group than in the control group (82/109, 55.0% vs 68/99, 45.0%; RR 1.10, 95% CI 0.92-1.30). Similar to this, at the final visit at 12 months, there was a greater percentage of participants with a diastolic BP of <85 mm Hg in the intervention group than in the control group (44/54, 55.0% vs 37/52, 45.0%; RR 1.15, 95% CI 0.92-1.42). These results failed to reach a statistically significant level (refer to Table 3).

**Table 3:** Diastolic blood pressure.

Diastolic blood pressure (mmHg)		Intervention group, n (%)	Control group, n (%)	Risk ratio (95% CI)	*P* value (overall)
**Baseline results (N=310)^a^**					.84
	<85	109 (51.0)	106 (49.0)	1.02 (0.88-1.18)	
	>85	46 (49.0)	49 (51.0)	1.02 (0.88-1.18)	
**6-month results (N=208)^b^**					.29
	<85	82 (55.0)	68 (45.0)	1.10 (0.92-1.30)	
	>85	27 (46.0)	31 (54.0)	1.10 (0.92-1.30)	
**12-month results (N=106)^c^**					.21
	<85	44 (55.0)	37 (45.0)	1.15 (0.92-1.42)	
	>85	10 (40.0)	15 (60.0)	1.15 (0.92-1.42)	

^a^Baseline: intervention group (n=155) and control group (n=155).

^b^6-month results: intervention group (n=109) and control group (n=99).

^c^12-month results: intervention group (n=54) and control group (n=52).

#### Glycosylated Hemoglobin A_1c_

At baseline, there was a smaller percentage of participants with an HbA_1c_ level <7% in the intervention group than in the control group (88/155, 45.0% vs 105/155, 55.0%). At the 6-month visit, there was a slightly higher percentage of stroke survivors with a HbA_1c_ level <7% in the intervention group than in the control group, (81/105, 52.0% vs 74/91, 48.0%; RR 0.95, 95% CI 0.82-1.10). Similarly, at the final visit at 12 months, there was a greater percentage of participants with an HbA_1c_ level <7% in the intervention group than in the control group (36/55, 55.0% vs 30/40, 45.0%; RR 0.87, with 95% CI 0.66-1.14). However, these results failed to demonstrate statistical significance (refer to Table 4).

**Table 4:** Glycosylated hemoglobin A_1c_.

Glycosylated hemoglobin A_1c_ (%)		Intervention group, n (%)	Control group, n (%)	Risk ratio (95% CI)	*P* value (overall)
**Baseline results (N=310)^a^**					.03
	<7	85 (45.0)	105 (55.0)	0.81 (0.68-0.98)	
	>7	70 (58.0)	50 (42.0)	0.81 (0.68-0.98)	
**6-month results (N=196)^b^**					.47
	<7	81 (52.0)	74 (48.0)	0.95 (0.82-1.10)	
	>7	24 (58.0)	17 (42.0)	0.95 (0.82-1.10)	
**12-month results (N=95)^c^**					.32
	<7	36 (55.0)	30 (45.0.0)	0.87 (0.66-1.14)	
	>7	19 (65.0)	10 (35.0)	0.87 (0.66-1.14)	

^a^Baseline: intervention group (n=155) and control group (n=155).

^b^6-month results: intervention group (n=105) and control group (n=91).

^c^12-month results: intervention group (n=55) and control group (n=40).

#### Low-Density Lipoprotein

At baseline, there was a greater percentage of study participants with an LDL level<100 mg/dL in the intervention group than in the control group (92/155, 59.0% vs 64/155, 41.0%). A similar trend was seen at the third follow-up visit: There was a higher percentage of stroke survivors with an LDL level<100 mg/dL in the intervention group than in the control group (73/106, 57.0% vs 56/90, 43.0%; RR 1.10, 95% CI 0.90-1.36). Again, a very similar trend was seen at the fifth follow-up visit: There was a greater percentage of participants with an LDL level <100 mg/dL in the intervention group than in the control group (36/51, 55.0%, vs 30/45, 45.0%; RR 1.06, 95% CI 0.81-1.39). However, these results failed to demonstrate statistical significance (refer to Table 5).

**Table 5:** Low-density lipoprotein.

Low-density lipoprotein (mg/dL)		Intervention group, n (%)	Control group, n (%)	Risk ratio (95% CI)	*P* value (overall)
**Baseline results (N=310)^a^**					<.001
	<100	92 (59.0)	64 (41.0)	1.42 (1.13-1.78)	
	>100	63 (41.0)	91 (59.0)	1.42 (1.13-1.78)	
**6-month results (N=196)^b^**					.33
	<100	73 (57.0)	56 (43.0)	1.10 (0.90-1.36)	
	>100	33 (49.0)	34 (51.0)	1.10 (0.90-1.36)	
**12-month results (N=96)^c^**					.68
	<100	36 (55.0)	30 (45.0)	1.06 (0.81-1.39)	
	>100	15 (50.0)	15 (50.0)	1.06 (0.81-1.39)	

^a^Baseline: intervention group (n=155) and control group (n=155).

^b^6-month results: intervention group (n=106) and control group (n=90).

^c^12-month results: intervention group (n=51) and control group (n=45).

### Secondary Outcomes

#### Mortality and the Number Needed to Treat

Overall, 35 deaths were reported over the course of 1-year follow-up in the Movies4Stroke trial (Multimedia Appendix 1). Mortality among stroke survivors because of stroke-related complications (included group) was higher in the control group than in the intervention group (13/155, 8.4% vs 2/155, 1.3%). The most common cause of mortality was aspiration pneumonia.

**Figure 4:**
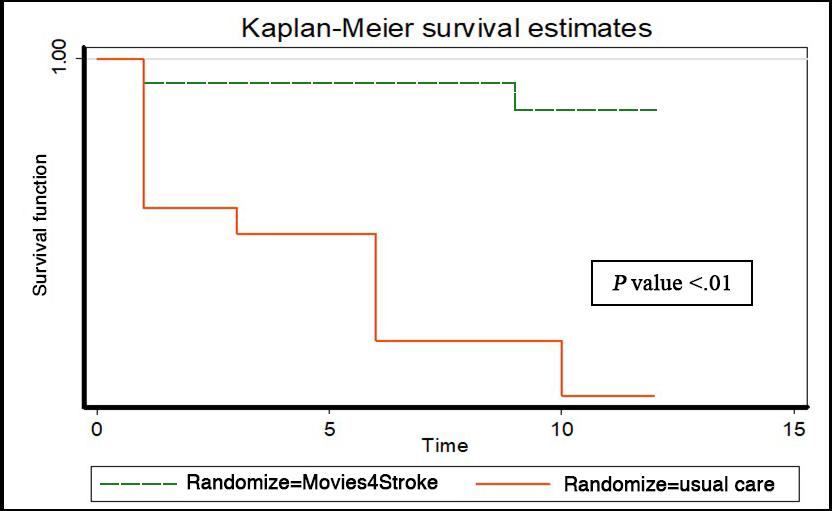
The Kaplan-Meier survival estimates in the first year after stroke in the intervention versus control group because of stroke-related avoidable mortality.

Censored deaths in both the groups were caused by non–stroke-related complications and were therefore not considered a part of the included group. The results were found to be highly significant (*P*<.001). Absolute risk reduction (ARR) of mortality related to stroke-related complications was 7%, which yielded a number needed to treat (NNT) of 15. It meant that we needed to show the video-based intervention to 15 stroke survivors to prevent 1 death from stroke-related complications (refer to Table 6 and Figure 4).

**Table 6:** Categorization of mortality, ARR, and NNT.

Mortality categorized	Intervention group (N=155), n (%)	Control group (N=155), n (%)	Absolute risk reduction	Number needed to treat	*P* value
Patient is alive	140 (90.3)	135 (87.1)	N/A^a^	N/A	N/A
Included	2 (1.3)	13 (8.4)	7.1	15^b^	<.001
Censored	10 (6.5)	4 (2.6)	N/A	N/A	N/A
Excluded	3 (1.9)	3 (1.9)	N/A	N/A	N/A

^a^Not applicable.

^b^Number needed to treat of 15 has been rounded off to a whole number.

### Change in Functional Status (Disability and Severity) Among Stroke Survivors

#### Modified Rankin Scale

**Figure 5:**
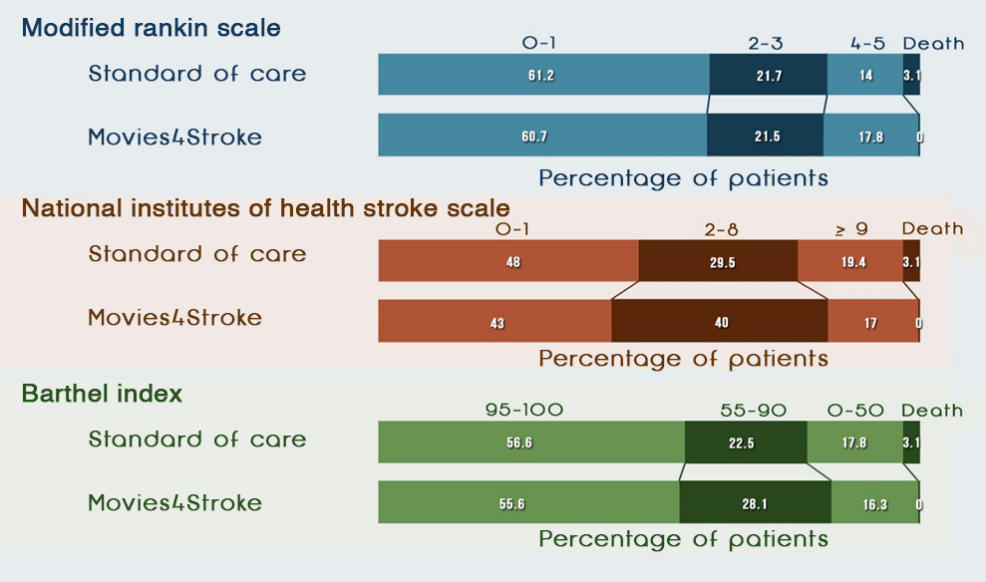
Functional status of stroke survivors—6 months. NIHSS: National Institutes of Health Stroke Scale.

**Figure 6:**
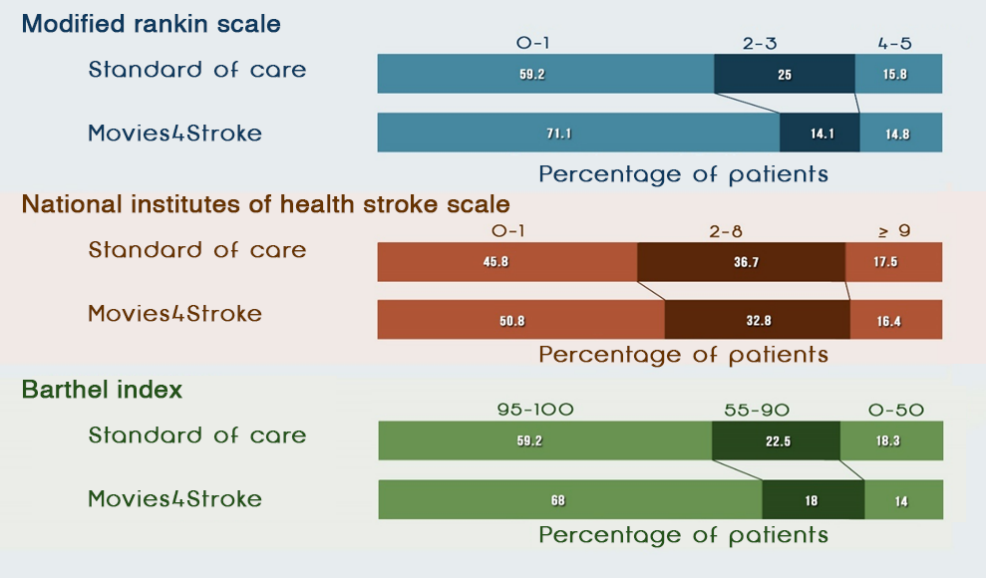
Functional status of stroke survivors—baseline and 12 months assessment (comparison).

Table 7 and Figures 5 and 6 show that at baseline, as assessed by mRS, a higher percentage of stroke survivors with moderate to severe disability were present in the intervention group than in the control group (46/155, 29.7% vs 36/155, 23.2%; odds ratio [OR] 1.18, 95% CI 0.58-2.39). Similarly, at 6 months, a higher percentage of stroke survivors with moderate to severe disability were present in the intervention group than in the control group (24/135, 17.8% vs 18/129, 14.0%; OR 1.28, 95% CI 0.65-2.55). However, at 12 months, a higher percentage of stroke survivors had minimal to no disability in the intervention group than in the control group (91/128, 71.1% vs 71/120, 59.2%). At 12 months postdischarge, NNT was 9, as assessed by the mRS. This meant that we needed to show Movies4Stroke to 9 stroke survivors to achieve minimal to no disability caused by stroke after a year of follow-up.

**Table 7:** Modified Rankin Scale.

Modified Rankin Scale score		Intervention group, n (%)	Control group, n (%)	Odds ratio (95% CI)	*P* value (overall)
**Baseline results (N=310)^a^**					.40
	0-1	25 (16.1)	25 (16.1)	Reference	
	2-3	84 (54.2)	94 (60.7)	0.82 (0.44-1.55)	
	4-5	46 (29.7)	36 (23.2)	1.18 (0.58-2.39)	
**6-month results (N=264)^b^**					.40
	0-1	82 (60.7)	79 (61.2)	Reference	
	2-3	29 (21.5)	28 (21.7)	0.99 (0.55-1.83)	
	4-5	24 (17.8)	18 (14.0)	1.28 (0.65-2.55)	
	Death	0 (0.0)	4 (3.1)	—^c^	
**12-month results (N=248)^d^**					.07
	0-1	91 (71.1)	71 (59.2)	Reference	
	2-3	18 (14.1)	30 (25.0)	0.47 (0.24-0.91)	
	4-5	19 (14.8)	19 (15.8)	0.78 (0.38-1.58)	

^a^Baseline: intervention group (n=155) and control group (n=155).

^b^6-month results: intervention group (n=135) and control group (n=129).

^c^Odds ratio with their 95% CI could not be estimated because of the empty cell count in the intervention group.

^d^12-month results: intervention group (n=128) and control group (n=120).

#### National Institutes of Health Stroke Scale

Table 8 and Figures 5 and 6 show that at baseline, as assessed by the NIHSS, a higher percentage of stroke survivors had severe impairment caused by stroke in the control group than in the intervention group (72/155, 46.4% vs 61/155, 39.4%; OR 0.82, 95% CI 0.39-1.74). At 6 months, a smaller percentage of stroke survivors had severe impairment caused by stroke in the intervention group than in the control group (23/135, 17.0% vs 25/129, 19.4%; OR 0.98, 95% CI 0.50-1.92). At 12 months, a higher percentage of stroke survivors had minimal to no impairment caused by stroke in the intervention group than in the control group (65/128, 50.8% vs 55/120, 45.8%). At 12 months postdischarge, NNT was 20 as per the NIHSS assessment. This meant that we needed to show Movies4Stroke to 20 stroke survivors to have minimal to no disability caused by stroke after a year of follow-up.

**Table 8:** National Institutes of Health Stroke Scale.

National Institutes of Health Stroke Scale score		Intervention group, n (%)	Control group, n (%)	Odds ratio (95% CI)	*P* value (overall)
**Baseline results (N=310)^a^**					.52
	0-1	18 (11.6)	17 (11.0)	Reference	
	2-8	76 (49.0)	66 (42.6)	1.09 (0.51-2.28)	
	>9	61 (39.4)	72 (46.4)	0.82 (0.39-1.74)	
**6-month results (N=264)^b^**					.08
	0-1	58 (43.0)	62 (48.0)	Reference	
	2-8	54 (40.0)	38 (29.5)	1.52 (0.88-2.63)	
	>9	23 (17.0)	25 (19.4)	0.98 (0.50-1.92)	
	Death	0 (0.0)	4 (3.1)	—^c^	
**12-month results (N=248)^d^**					.73
	0-1	65 (50.8)	55 (45.8)	Reference	
	2-8	42 (32.8)	44 (36.7)	0.81 (0.46-1.41)	
	>9	21 (16.4)	21 (17.5)	0.85 (0.42-1.71)	

^a^Baseline: intervention group (n=155) and control group (n=155).

^b^6-month results: intervention group (n=135) and control group (n=129).

^c^Odds ratio with their 95% CI could not be estimated because of the empty cell count in the intervention group.

^d^12-month results: intervention group (n=128) and control group (n=120).

#### Barthel Index

Table 9 and Figures 5 and 6 show that at baseline, as assessed by the BI, an equal percentage of stroke survivors had total to severe dependency in the intervention group as compared with the control group (78/155, 50.3% vs 78/155, 50.3%; OR 1.08, 95% CI 0.63-1.86). At 6 months, a smaller percentage of stroke survivors had total to severe dependency in the intervention group than in the control group (22/135, 16.3% vs 23/129, 17.8%; OR 0.93, 95% CI 0.48-1.81). At 12 months, a higher percentage of stroke survivors with minimal to no dependency were present in the intervention group than in the control group (87/128, 68.0% vs 71/120, 59.2%). At 12 months postdischarge, NNT was 12, as per the BI assessment.

**Figure 7:**
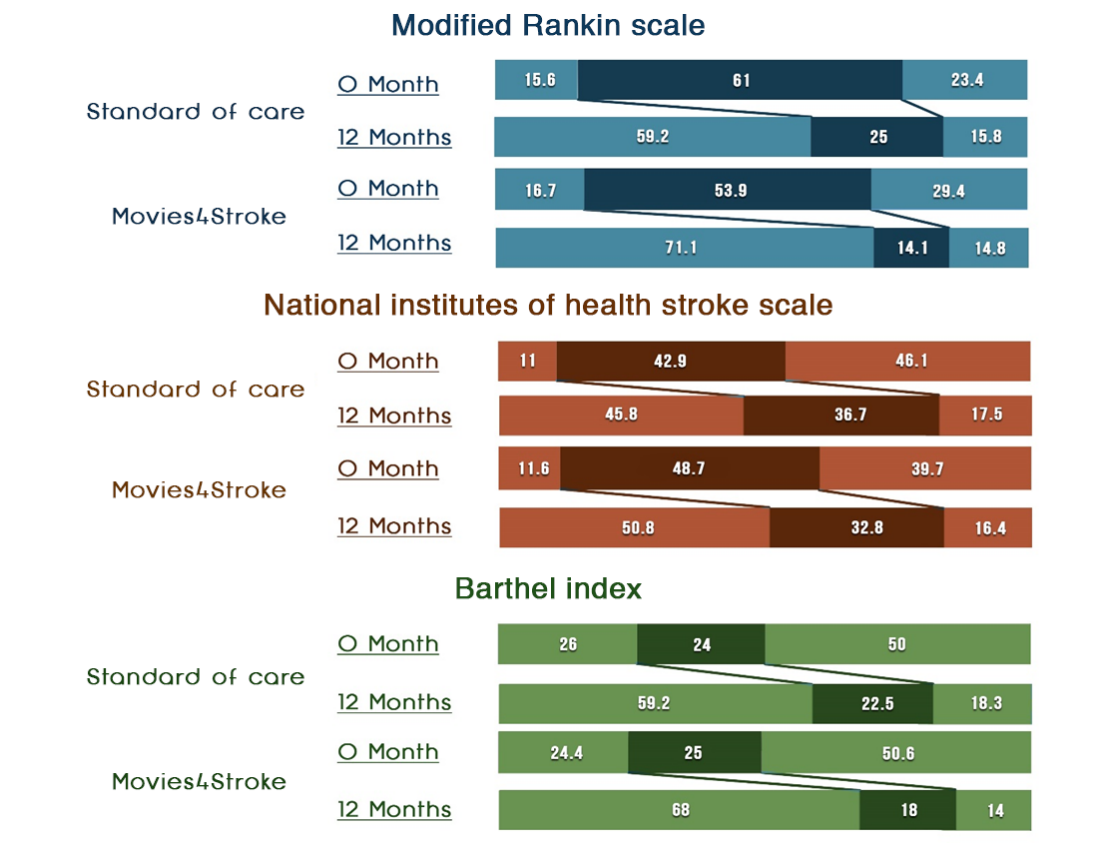
Functional status of stroke survivors—baseline and 12 months assessment (comparison).

As evident in Figure 7 and according to the mRS, when comparing the baseline with the 12-month follow-up visit, more survivors in the intervention group had seen the videos and had minimal to no disability at the end of the 12 months as compared with the control group. Similarly, according to NIHSS, when comparing baseline with 12-month follow-up visits, there was a higher percentage of survivors with minimal neurologic deficit at 12 months in the intervention group as compared with the control group. Similarly, for BI, when comparing baseline with 12-month follow-up visits, there was a higher percentage of survivors with had minimal to no dependency at 12 months in the intervention group as compared with the control group.

**Table 9:** Barthel Index.

Barthel Index score		Intervention group, n (%)	Control group, n (%)	Odds ratio (95% CI)	*P* value (overall)
**Baseline results (N=310)^a^**					.94
	95-100	38 (24.5)	40 (25.8)	Reference	
	55-90	39 (25.2)	37 (23.9)	1.11 (0.59-2.09)	
	0-50	78 (50.3)	78 (50.3)	1.08 (0.63-1.86)	
**6-month results (N=264)^b^**					.16
	95-100	75 (55.6)	73 (56.6)	Reference	
	55-90	38 (28.1)	29 (22.5)	1.28 (0.71-2.28)	
	0-50	22 (16.3)	23 (17.8)	0.93 (0.48-1.81)	
	Death	0 (0.0)	4 (3.1)	—^c^	
**12-month results (N=248)^d^**					.35
	95-100	87 (68.0)	71 (59.2)	Reference	
	55-90	23 (18.0)	27 (22.5)	0.70 (0.37-1.32)	
	0-50	18 (14.0)	22 (18.3)	0.67 (0.33-1.34)	

^a^Baseline: intervention group (n=155) and control group (n=155).

^b^6-month results: intervention group (n=135) and control group (n=129).

^c^Odds ratio with their 95% CI could not be estimated because of the empty cell count in the intervention group.

^d^12-month results: intervention group (n=128) and control group (n=120).

## Discussion

### Principal Findings and Their Contextual Relevance

Movies4Stroke is a randomized controlled mHealth trial evaluating the effectiveness and safety of a phone-based intervention that showed thematically designed videos to assist the stroke survivor and caregiver dyad to get the knowledge, skills, and confidence needed to improve poststroke risk factor control, survival, and functional outcomes. The rationale of this trial was to provide repetitive high-quality survival and training to stroke survivor and caregiver dyads returning to a low- to middle-income community where rehabilitation and chronic care systems are underdeveloped. The mode of video-based IT intervention, along with competency and understanding checks and the ability to repeat and see a video, was used to assist the understanding in a literacy-challenged population that, however, had access to a cell phone. None of the trial participants were excluded because of the lack of a cell phone. This mHealth trial failed to reach its primary outcome measure of the control of hypertension, LDL cholesterol, and HbA_1c_; however, the prespecified secondary variables of improved functional outcomes and reduced mortality were improved in the intervention group in this study because of stroke-related complications. This study demonstrated that showing the video to a literacy-challenged population in a country without any chronic care health facility saved lives. The NNT was only 15, to save 1 life, because of avoidable stroke-related complications and death. This trial provides early data on nonpharmacological intervention in stroke survivors. There are few interventions of this nature identified in a recent meta-analysis and review and none that employs mHealth in a complex, challenging, and resource-strapped setting (refer to Multimedia Appendix 2) [15-21].

### Primary and Secondary Outcome Results: Mechanistic Explanations

Movies4Stroke failed to achieve its primary outcome variable of the control of primary risk variables of hypertension, LDL cholesterol, or HbA_1c_ in the intervention. There could be several reasons for these findings. First, the baseline adherence in this population was already substantially better than the general population, possibly because of the seriousness of stroke and exposure to specialist care and counseling techniques; thus, it became difficult to further improve adherence and the related improvement in risk factors. This could be explained by the Health Belief Model demonstrating that better outcomes are found in patients who understand the severity of their disease and the benefits that changing their actions would yield [22,23].

Second, in this study, to maintain long-term follow-up, the control group also had access to a 24/7 helpline, and interaction with health care personnel who assisted and resolved queries may have improved behavior in this group as well. The presence of a centralized helpline has shown to help address queries and patients cope with difficult situations [24]. 

Third, this was a sequential and thematic intervention where these 5-min video lessons were rolled out in order of what skill would be important for survival in the early stages as compared with the later stages of stroke. Hence at admission, the survivor and caregiver dyad were provided with skills and training to recognize life-threatening emergencies and respond to them accordingly. At the time of discharge, each dyad was taught rehabilitation and safe swallowing. Thematic movies on adherence and medication safety module were taught in the first month; thus, these preventive aspects were dealt with much later, and this might in itself be associated with learning fatigue, or perhaps, these movies were taught by the time the risk factor control had already maximized in the participants in the study.

Finally, it is difficult to design measures that capture pill compliance in the stroke population; stroke patients have diverse prescriptions that vary in the type of drug classes, number, and frequency of dosage, and no single biomarker would be applicable to all the study participants nor could electronic pill boxes capture the actual drug usage to achieve BP, glucose, or cholesterol control, as they record the number of times a box is opened. Moreover, it cannot be assumed that the participants have consumed all the pills for that dose when they open a box. However, we can see that the harder outcome measure of the control of BP, cholesterol, or blood sugar was not achieved.

In addition, these negative findings on risk factor control shed light on the importance of health theory and behavioral science designing an intervention, rather than considering just simple knowledge transfer while designing an intervention. This resonates with previous studies concluding that to achieve effective change in health systems and patient care, knowledge transfer alone is insufficient and has to be supplemented with other forms of intervention [25]. In a single-center study [26], showing just one video in an in-patient setting increased knowledge; however, it did not translate into BP monitoring in a home environment or increase physician follow-up visits. The sustained intervention in our study revealed that, at least the skills to prevent complications might have been acquired.

Nonadherence to medication has two components—intentional and unintentional nonadherence [27]. Intentional nonadherence refers to nonadherence that is deliberate and may be because of motivational factors that may be directly related to the perceived efficacy of the medicine, distrust, and the lack of knowledge, whereas unintentional nonadherence is nonadherence that is largely related to either forgetting or the lack of capacity to take the medicine. In our study, we focused on improving knowledge, empowerment, and self-efficacy, but we did not send repeated reminders to improve unintentional nonadherence. Similar results were obtained in a study implemented in Sydney, Australia, focusing on participants with coronary heart disease in which repeated lifestyle modification messages/reminders showed significant results with respect to harder outcomes such BP and cholesterol [28]. This highlights the importance of mHealth design to be multifaceted and used as a platform for re-enforcement to address behavior change with regard to adherence. It also highlights the potential of mHealth to be harnessed in reducing the burden of NCDs when interventions are designed using health theory, and they can inform effectiveness, thus achieving the millennium development goals [29,30].

Interestingly, each of our prespecified secondary outcome variables improved in this study. Secondary outcomes of Movies4Stroke trial aimed at improving functional outcomes and reducing mortality because of stroke-related complications, and the results are very promising, given the difficult context of the intervention. The mortality analysis showed that in the control group, there were 9 of 13 cases of massive aspiration pneumonias that resulted in mortality, which could have been avoided by aspiration precautions and learning tube feeding safety measures. In addition, recognizing aspiration and reporting early could have resulted in saving lives. There were 2 mortalities in the intervention group, one because of a massive recurrent stroke and the other because of aspiration. The other mortalities were not related directly to stroke-mediated complications. Given the limited nature of these observations, these findings are worthy of attention but need to be confirmed in larger cohorts with prolonged follow-up periods.

Skills to help identify, prevent, and respond to poststroke complications were taught through 5-min videos that had been rolled out in an orderly manner, with preventable early complications addressed first, followed by late complications. This method of interactive, repeated teaching increased the skill and confidence of the caregivers and thus resulted in saving lives and improving the functional outcomes and reducing disability.

Other studies have similarly reported that timely recognition and reporting of complications resulted in low mortality and improved outcomes among stroke survivors [31,32]. Through these movies, knowledge transfer helped change the skills of the caregivers rather than the adherence of stroke survivors. Along with this, Movies4Stroke might have influenced health beliefs of stroke survivors and their caregivers by helping them understand their increased susceptibility and therefore the importance of prompt action to prevent death because of complications [33,34]. Moreover, Movies4Stroke also had videos aimed at providing psychoeducational intervention and support through the helpline to the caregivers along with the procedural knowledge. These three components have been pivotal in helping prevent stress and strain in informal caregivers [19], which may also explain the active involvement and effective skill knowledge of caregivers in this study.

Owing to the varying definitions of successful stroke recovery, it is difficult to find a standard measure of recovery, which is why we used three different scales [35]. The improvement observed in functional outcomes in the intervention group might have been because of the learning model that these videos were based upon. Rehabilitation measures and approaches were described and taught in the videos. However, the demonstration of each, by registered professional physiotherapists, nurses, stroke survivors with significant disability, and speech and swallowing experts, helped augment the learning process and made it easy for patients and their caregivers to mimic and follow each step taught effectively. One of the most important aspects of these movies was watching real stroke survivors perform the exercises themselves; it acted as a morale booster and further encouraged participants to continue with the exercises. The added feature of being able to replay and rewatch these videos might have been another factor that aided their learning process, resulting in significant improvement in functional status.

These results are encouraging in several ways. First, as the extent of improvement in the functionality of stroke survivors demonstrates the ability to return to an improved functionality level with the help of only these movies and their primary caregivers without the need of a skilled nurse or a standard rehabilitation center. This is similar to the results of a trial that compared and concluded equivalent improvements in patients who received home-based rehabilitation therapy with a caregiver and others who received therapy with a professional therapist, thereby establishing home-based rehabilitation as equally effective, as shown with Movies4Stroke [36]. These video lessons filled the niche that the lack of these interventions might have created and bridged the gap between learning and implementation process [37]. Although there have been several discussions calling for attention to NCDs at national and international level to help meet the MDGs, there has also been concern regarding the high costs of treatment and recurrence of debilitating events such as cardiovascular death. These have prompted debate among the policy makers to establish ways for primary prevention, affordable treatment measures, and monitoring of NCDs [38,39]. Movies4Stroke is a step in this direction, aimed at providing better health care, and prevention through diet and healthy activity, thus curbing the alarming burden of NCDs in LMICs

### Trial Strengths

The major strengths of this trial were the use of a randomized controlled trial design at a center where an internationally certified care model is followed with an algorithmic approach; hence, the results generated can be attributed to the effect of intervention.

The risk of bias was minimized by paying attention to domains that defined trial quality [19,40,41]. Selection bias was reduced by random centralized computer-based sequence generation; allocation was concealed by opaque sequentially numbered envelopes that were dispatched by CTU, which included university-based staff, which was separate from the research team. Performance and detection bias was reduced by blinded outcome assessment and cross-checks of contamination during the course of the trial and uniform training of staff on study. Attrition bias was addressed by multiple means of follow-up—maintaining good level of understanding with the participants, sending SMS reminder for follow-ups, and preserving communication through Stroke Helpline number. We were successful in keeping an overall dropout rate of less than 9%, with sensitivity analysis showing that were no significant differences between the characteristics of dropouts/those lost to follow-up and those who continued the study in both the groups (Multimedia Appendix 3). Reporting bias was reduced by reporting all possible outcomes of interest as defined in the protocol. Intention-to-treat analysis has been used to report outcomes.

To ensure compliance to the intervention, the first time, the videos were viewed in the presence of a research officer, and intervention was delivered at 100% rate, with complete verbalized understanding and unhurried visualization. To track usage patterns at home, we remotely monitored the access and use of our app that had been installed in the mobile phone. Early results show that the rehabilitation and tube feeding videos were the highly accessed videos at home. These analyses are still ongoing.

### Trial Limitations

The major limitation of our clinical trial is that it is a single-center study, chosen because of the fact that this site provided a standard of care that is algorithmic and replicable; thus, results generated can be attributed to the effect of intervention. In this study, we reported its efficacy, but the performance and potential effect size in different sites may be variable and potentially be more effective, given the usual care standards in even more resource-strapped comparative health systems, for example, the government sector, and thus, external validity is limited. Furthermore, those with more severe stroke or better health access because of socioeconomic status are more likely to visit the health systems and volunteer to participate in an educational training intervention, thus limiting direct external validity. We would definitely include nonresponse analysis in futures studies to further identify characteristics of those most likely to adopt the intervention. Another significant limitation is of contamination bias in an educational intervention. Care was taken to avoid contamination of the nonintervention group with the intervention. To ensure this, videos were shown in a separate room and not at the bedside. Given the fact that families share information, contamination was possible; however, most stroke survivors were on different schedules for follow-up visits and rehabilitation times, and so, we expected less contamination than in areas where a lot of time is spent together by families. Obviously, it is an inherent limitation of an educational intervention that blinding of participants is not possible. This intervention in a study setting required human resources in terms of a study officer and IT back up, development required human resource, and deployment required at least three staff in a study setting. To predict feasibility, we need to further analyze cost-effectiveness and realistic compliance in a clinical setting with the current patient volumes, so this limits directly recommending applicability. This study has collected data on cost-effectiveness, which is under analysis at this time. 

### Way Forward

In conclusion, we demonstrate the potential of mHealth interventions to save lives and reduce disability in low- to middle-income country settings; we also show that these interventions are safe and feasible, despite their complexity. Our results demonstrate the key importance of health theory in designing these complex health interventions for replicability and informing further interventions. Interventions that target compliance must have repetitive reminders for nonintentional adherence, and those that target knowledge and skills transfer must have the capacity to repeat and bolster confidence as well as provide the user the opportunity to model themselves from the materials taught, despite the lack of literacy skills to be safe. Complex interventions targeting these settings need to have a design theory in place to deliver both these aspects to be effective.

Please refer to Consolidated Standards of Reporting Trials-eHealth checklist for details regarding this Movies4Stroke trial [42].

## Appendix

Multimedia Appendix 1 Detailed Mortality Analysis.

Multimedia Appendix 2 Non-pharmacological interventions targeting stroke survivors and their caregivers.

Multimedia Appendix 3 Analysis of Lost to Follow Up and Mortality.

Multimedia Appendix 4 CONSORT-EHEALTH checklist V1.6.1.

## Abbreviations

AKUH: Aga Khan University Hospital
ARR: absolute risk reduction
BI: Barthel Index
BP: blood pressure
CTU: clinical trials unit
eHealth: electronic health
ERC: Ethical Review Committee
HbA_1c_: glycosylated hemoglobin A_1c_
IT: information technology
LDL: low-density lipoprotein
mHealth: mobile health
mRS: Modified Rankin Scale
NCD: noncommunicable disease
NIHSS: National Institutes of Health Stroke Scale
NNT: number needed to treat
OR: odds ratio
RR: risk ratio
