# Supplementary material for: Effect of 5-Minute Movies Shown via a Mobile Phone App on Risk Factors and Mortality After Stroke in a Low- to Middle-Income Country: Randomized Controlled Trial for the Stroke Caregiver Dyad Education Intervention (Movies4Stroke)
Source: JMIR Mhealth Uhealth. 2020 Jan 28;8(1):e12113. doi: 10.2196/12113 (PMC7013656; doi:10.2196/12113)
Supplement: Multimedia Appendix 1 [file mhealth_v8i1e12113_app1.docx]

**Appendix 2: Detailed analysis of Mortality according to the criteria of Included, Excluded and Censored deaths**

| **S. No** | **Study Id.** | **M.R No.** | **Study Arm** | **Mortality Taken As** | **Description** | **Cause Of Death** | **Case Ascertainment** |
| --- | --- | --- | --- | --- | --- | --- | --- |
| 01 | 012 | 197-70-94 | Control | Included | Readmission (In-Hospital Mortality) | - Aspiration Pneumonia - Acute Kidney Infection | Hospital Records And Verbal Autopsy |
| 02 | 017 | 179-55-83 | Intervention | Censored | Out Of Hospital Mortality | - Chronic Kidney Disease - Silent MI | Verbal Autopsy |
| 03 | 027 | 197-73-68 | Control | Included | Readmission (In-Hospital Mortality) | - Aspiration Pneumonia - ↑C-Reactive Protein suspected of Infection and Sepsis - Upper G.I. Bleeding - Heart Attack (Emergency in Ambulance) | Hospital Records And Verbal Autopsy |
| 04 | 034 | 197-75-47 | Intervention | Excluded | In-Hospital Mortality | - Malignant MCA Infarction - Pneumonia - Huge CVA | Medical Records |
| 05 | 039 | 197-76-87 | Control | Included | Out Of Hospital Mortality | - Recurrent Intra Cranial Haemorrhage (3months) | Verbal Autopsy |
| 06 | 055 | 197-79-89 | Control | Included | Out Of Hospital Mortality | - Aspiration Pneumonia - Chest Complication | Verbal Autopsy |
| 07 | 063 | 026-64-51 | Control | Included | Out Of Hospital Mortality | - Sudden Breathing Difficulty - Sudden Onset of fever - Aspiration Pneumonia - Patient got distressed and his condition worsened. | Hospital Records And Verbal Autopsy |
| 08 | 065 | 197-83-86 | Intervention | Excluded | In-Hospital Mortality | - Massive Left MCA Infarct - DNR Pharma - CKD - HTN   (IHD & EF = 25%)   - Bradycardia - Asystole | Hospital Records |
| 09 | 073 | 268-70-47 | Intervention | Censored | Out of Hospital Mortality | - Generalized weakness and development of Bed sores. - Cause of Death Unknown - Probable cause- Sudden Cardiac death | Verbal Autopsy |
| 10 | 077 | 197-88-04 | Intervention | Censored | Out of Hospital Mortality | - Patient came on the same day in morning clinic for usual follow-up. - Patient went home, was feeling feverish and had mild discomfort - Sudden increase in Blood Pressure - Sudden Cardiac Death | Verbal Autopsy |
| 11 | 079 | 197-87-63 | Control | Excluded | In-Hospital Mortality | - Left BG Bleed - Septic Shock - Intubated - AKI | Hospital Records |
| 12 | 086 | 164-89-18 | Intervention | Censored | Out-Of Hospital Mortality | - Left MCA - Subdural Hematoma (Trauma) - LAMA Discharge - (Died After 13 Days) | Hospital Records And Verbal Autopsy |
| 13 | 103 | 009-43-50 | Intervention | Censored | Out-Of Hospital Mortality | - EF = 25% - Large Massive MI - Heart Failure | Hospital Records And Verbal Autopsy |
| 14 | 105 | 110-41-90 | Control | Excluded | In-Hospital Mortality | - BG Bleed - Unstable Stroke - Fast AFib - Sudden BP Dropped | Hospital Records |
| 15 | 141 | 033-88-05 | Intervention | Censored | In Hospital Mortality | - Two vessel coronary artery disease with 100% occlusion of RCA and LAD - Ejection Fraction 20% - Heart Attack | Hospital Records and Verbal Autopsy |
| 16 | 145 | 197-99-32 | Control | Censored | In Hospital Mortality | - 30% narrowing of   the lumen in the right internal carotid artery.   - 20% narrowing of   the lumen in the left internal carotid artery.   - Severe chest pain   since 4-5 days   - Admitted to NICVD - Diagnosed with   Myocardial Infarction   - Experienced   shortness of breath and congestion   - Couldn’t survive for   long | Verbal Autopsy |
| 17 | 148 | 198-05-77 | Control | Included | Out-Of Hospital Mortality | - Aspiration pneumonia - Meningitis | Hospital Records And Verbal Autopsy |
| 18 | 160 | 198-10-30 | Control | Included | In-Hospital Mortality | - Recurrent Stroke - Renal Failure - Advised Dialysis - Died due to complications of Renal Failure | Hospital Records And Verbal Autopsy |
| 19 | 165 | 513-40-16 | Control | Censored | Out-Of Hospital Mortality | - EF = 15-20% - AKI - Pneumonia - Died In Sleep | Hospital Records And Verbal Autopsy |
| 20 | 166 | 198-11-24 | Intervention | Excluded | In-Hospital Mortality | - Transferred To Another Facility Before Being Discharged - Hospital Acquired Pneumonia - Dead | Hospital Records And Verbal Autopsy |
| 21 | 171 | 198-13-66 | Control | Included | Out-Of Hospital Mortality | - Recurrent CVA - Aspiration Pneumonia - AFib - AKI - NG Tube Feeding Complications | Hospital Records And Verbal Autopsy |
| 22 | 179 | 610-87-20 | Control | Censored | Out-Of Hospital Mortality | - Diabetes - Fungal Infection - LAMA | Hospital Records And Verbal Autopsy |
| 23 | 201 | 270-09-34 | Intervention | Included | Out-Of Hospital Mortality | - Left MCA Infarction - Heart Attack In Ambulance - Sudden MI & EF = 55% - Systolic Dysfunction | Verbal Autopsy |
| 24 | 210 | 198-28-90 | Control | Included | In-Hospital Mortality | - Recurrent Stroke - Aspiration Pneumonia - Chest Pain - Congestion | Verbal Autopsy And Hospital Records |
| 25 | 219 | 260-31-30 | Control | Included | Out-of Hospital Mortality | - Massive attack of stroke (Fatal Stroke ) | Hospital Records And Verbal Autopsy |
| 26 | 221 | 198-31-51 | Intervention | Included | In Hospital Mortality | - Shortness of Breath - LAMA - Shifted to another hospital - Diagnosed with Aspiration Pneumonia - Condition worsened and patient couldn’t survive | Hospital Records and Verbal Autopsy |
| 27 | 228 | 197-62-21 | Intervention | Censored | Out-of Hospital Mortality | - Died due to heat stroke | Hospital Records And Verbal Autopsy |
| 28 | 257 | 198-43-97 | Control | Included | Out-of Hospital Mortality | - Aspiration pneumonia | Hospital Records And Verbal Autopsy |
| 29 | 258 | 198-43-93 | Intervention | Censored | Out-of Hospital Mortality | - TB Meningitis - Right MCA Territory Infarct - LAMA | Hospital Records And Verbal Autopsy |
| 30 | 259 | 223-15-81 | Intervention | Censored | Out-of Hospital Mortality | Sudden cardiac death due to pre-existing IHD | Hospital Records And Verbal Autopsy |
| 31 | 261 | 194-81-69 | Control | Excluded | In-Hospital Mortality | - Left MCA Infarct - Atrial Fibrillation - RVR - Aspiration Pneumonia | Hospital Records And Verbal Autopsy |
| 32 | 266 | 198-47-66 | Intervention | Censored | Out-of Hospital Mortality | - Kidney failure - Atrial Fibrillation - Lacto acidosis (electrolyte imbalance) - Cardiac Blockade | Hospital Records And Verbal Autopsy |
| 33 | 276 | 246-46-89 | Control | Censored | Out-of Hospital Mortality | - Dehydration - Sudden Myocardial Infarction | Verbal Autopsy |
| 34 | 285 | 198-61-05 | Control | Included | Out-of Hospital Mortality | - Aspiration Pneumonia with sepsis. | Hospital Records And Verbal Autopsy |
| 35 | 305 | 198-74-32 | Control | Included | Readmission  (In-Hospital Mortality) | - Recurrent Stroke - Hypertension - Vomiting | Hospital Records And Verbal Autopsy |

|  |  |  |  | **CASE ASCERTAINMENT** |  |
| --- | --- | --- | --- | --- | --- |
|  |  |  |  | Hospital records/Verbal Autopsy | |

**Analysis**

→ Mortality included in the Control: **13**

→ Mortality included in the Intervention: **2**

→ Mortality excluded from the Control: **3**

→ Mortality excluded from the Intervention: **3**

→ Mortality censored in the Control: **4**

→ Mortality censored in the Intervention: **10**

- Total number of deaths in Movies4Stroke Trial **= 35**
