# Supplementary material for: Effect of 5-Minute Movies Shown via a Mobile Phone App on Risk Factors and Mortality After Stroke in a Low- to Middle-Income Country: Randomized Controlled Trial for the Stroke Caregiver Dyad Education Intervention (Movies4Stroke)
Source: JMIR Mhealth Uhealth. 2020 Jan 28;8(1):e12113. doi: 10.2196/12113 (PMC7013656; doi:10.2196/12113)
Supplement: Multimedia Appendix 2 [file mhealth_v8i1e12113_app2.docx]

**Appendix 1:** Non-pharmacological interventions targeting stroke survivors and their caregivers

| **Study ID** | **Design** | **Participants** | **Duration** | **Intervention** | **Control** | **Intensity** | **Results** | **Conclusion** |
| --- | --- | --- | --- | --- | --- | --- | --- | --- |
| Lindley, 2017[15] | RCT | 1212 caregivers | 2 years | Evidence based rehabilitation with information provision, caregiver training by rehabilitation professionals starting from hospitalization till 2 months post-discharge | Standard stroke care | 1 hr x 3 days in hospital 6 home visits over 2 months Intervention manual to help caregivers continue patient rehabilitation at home | No significant difference | No conclusive evidence to encourage caregiver-based rehabilitation services, future research should focus on healthcare assistants or community care |
| Forster, 2013[16] | Cluster- RCT | 928 stroke survivor and caregiver dyads | 2 years | London Stroke Careers Training Course  (LSCTC) training given to stroke rehabilitation units to impart to stroke survivors and caregivers. | Standard stroke care | Outcomes assessed at 6 months and 12 months | No significant difference | Inpatient training delivery to caregivers needs augmentation by follow-up training post-discharge |
| Markle Reid, 2011 [17] | RCT | 101 stroke survivors | 1 year | Standard home care plus home visitation by an interprofessional team of care coordinator, registered nurse, physiotherapist, occupational therapist, dietitian, social worker and personal support worker. Stroke rehabilitation, stroke education, caregiver support, referral to health and social services was provided. | Usual homecare, with physiotherapists, nurses etc as needed | Outcomes assessed and compared between baseline and at 12 months | Significant improvement in primary outcome, none in secondary outcome | A specialized interprofessional team can improve quality of life through better community-based stroke rehabilitation than usual home care |
| Cameron, 2015[18] | RCT | 31 caregivers | 6 months | Two interventions: self-directed and stroke support directed. The latter offered support to caregivers and included in-person meeting during admission, followed by monthly phone calls to discuss caregiver wellbeing, caregiving strategies, patient well-being and service delivery. A guidebook was also provided that describes patients’ recovery phases and information to deal with them. | Standard care and a self –directed intervention. | Outcomes assessed at baseline, 1, 3 and 6 months. | Significant improvement in perceived support and mastery in stroke support intervention vs self-directed arm, and the latter vs control arm. | Support intervention will aid in improving caregiver’s mastery and perceived support. |
| Legg, 2011[19] | Review | 1007 caregivers | 44 years (1967-2011) | The effect of nonpharmacological interventions aimed at informal caregivers of stroke survivors or at the caregiver and stroke survivor dyads. | As per studies included | As per studies included | No significant improvement | Meta-analysis of results from RCTs is not possible because of clinical, methodological and statistical heterogeneity. |
| Chumbler, 2015[20] | RCT | 52 patients | 3 months | Intervention group offered standard home care with stroke telerehabilitation (STeleR) intervention. They offered three home visits, five telephone calls, and an in-home messaging device provided over three months to teach patients exercises and adaptive strategies. | Usual home care | Outcomes assessed at baseline, 3 and 6 months | No significant improvement in Falls Efficacy Scale but improvement in physical function and satisfaction with hospital care. | The STeleR intervention had a greater effect on hospital satisfaction than home satisfaction. |
