# Supplementary material for: Effect of 5-Minute Movies Shown via a Mobile Phone App on Risk Factors and Mortality After Stroke in a Low- to Middle-Income Country: Randomized Controlled Trial for the Stroke Caregiver Dyad Education Intervention (Movies4Stroke)
Source: JMIR Mhealth Uhealth. 2020 Jan 28;8(1):e12113. doi: 10.2196/12113 (PMC7013656; doi:10.2196/12113)
Supplement: Multimedia Appendix 3 [file mhealth_v8i1e12113_app3.docx]

**Appendix 3: Analysis of dropouts (lost to follow-up) and mortality at each Study Time Point – Movies4Stroke Trial**

**Lost to Follow-Up Only at each Study Time Point**

| **S.No** | **First Follow-up** | | **Second Follow-up** | | **Third Follow-up** | | **Fourth Follow-up** | | **Fifth Follow-up** | |
| --- | --- | --- | --- | --- | --- | --- | --- | --- | --- | --- |
| **Study Group** | Intervention  Group | Control  Group | Intervention  Group | Control  Group | Intervention  Group | Control  Group | Intervention  Group | Control  Group | Intervention  Group | Control  Group |
| **Number of Dropouts** | 4 | 7 | 1 | 3 | 2 | 4 | 0 | 0 | 4 | 1 |
| **Total** | 11 | | 04 | | 06 | | 00 | | 05 | |

Total number of dropouts till 12 month follow-up: **26**

Percentage of drop Outs: **8.38%**

Percentage intact (remained in the study): **91.62%**

**Lost to Follow-Up & Deaths at each Study Time Point**

| **S.No** | **First Follow-up** | | **Second Follow-up** | | **Third Follow-up** | | **Fourth Follow-up** | | **Fifth Follow-up** | |
| --- | --- | --- | --- | --- | --- | --- | --- | --- | --- | --- |
| **Study Group** | Intervention  Group | Control  Group | Intervention  Group | Control  Group | Intervention  Group | Control  Group | Intervention  Group | Control  Group | Intervention  Group | Control  Group |
| **Deaths** | 8 | 11 | 1 | 1 | 3 | 5 | 1 | 0 | 2 | 3 |
| **Drop outs** | 4 | 7 | 1 | 3 | 2 | 4 | 0 | 0 | 4 | 1 |
| **Total in each Group** | 12 | 18 | 2 | 4 | 5 | 9 | 1 | 0 | 6 | 4 |
| **Overall Total** | 30 | | 6 | | 14 | | 2 (One was protocol violation) | | 10 | |
| **Overall %age** | 9.67% | | 1.93% | | 4.52% | | 0.64% | | 3.22% | |

**PROTOCOL VIOLATION:**

**Number of Protocol Violation:** 01

**Study ID:** 233

**Reason:** Patient included in the study due to misdiagnosis (symptoms of TIA due to meningitis).
